# Medication Adherence Status and Influencing Factors in Adolescent Kidney Transplant Recipients

**DOI:** 10.1111/petr.70103

**Published:** 2025-06-05

**Authors:** Yanhua Li, Yutong Chen, Shijun Pu, Suxia Yang, Li Zeng, Yazhe Duan, Xiaoying Lu, Wenyu Zhao

**Affiliations:** ^1^ Department of Organ Transplantation The First Affiliated Hospital of Naval Medical University Shanghai China; ^2^ Department of Nursing The First Affiliated Hospital of Naval Medical University Shanghai China

**Keywords:** adolescent, influencing factors, kidney transplant, medication adherence, overall well‐being

## Abstract

**Background:**

This study investigated medication adherence and related factors in adolescent kidney transplant recipients receiving immunosuppressive drugs.

**Methods:**

Between February and October 2023, 115 adolescent kidney transplant recipients were selected through convenience sampling from follow‐up centers in four Chinese tertiary hospitals. The study assessed medication adherence and influencing factors via a general information questionnaire, an adherence assessment scale, a medication belief scale, and an overall happiness scale.

**Results:**

The average medication adherence score was 4.98 ± 2.32 points, with 60.08% (76/115) showing poor adherence. Five of these patients experienced graft failure, resulting in a 6.5% failure rate. No cases of graft failure occurred in those with excellent compliance. Univariate analysis revealed no significant differences in adherence based on sex, age, income, or education (*p* > 0.05). However, statistically significant differences in medication adherence were observed based on rejection reactions and infections (*p* < 0.01). Pearson's correlation analysis revealed that higher perceived medication necessity and overall happiness scores were correlated with better adherence (*p* < 0.05). Linear regression revealed that post‐transplant rejection, infections, perceived medication necessity, and overall happiness significantly impacted adherence (*p* < 0.05).

**Conclusion:**

The high prevalence of poor medication adherence among adolescent kidney transplant recipients highlights the importance for healthcare professionals to focus on monitoring significant post‐transplant complications, improving recipients' understanding of the necessity of their medications, and evaluating their overall psychological well‐being. Introducing an effective early warning system during home follow‐up periods can enable timely interventions to improve medication adherence.

## Graphical abstract

The findings of this study demonstrate that post‐transplant rejection, infections, perceived medication necessity, and overall happiness significantly influence medication adherence among adolescent kidney transplant recipients. It is therefore recommended to introduce an early warning intervention system during home follow‐ups, specifically designed to enhance medication adherence by addressing these critical factors.

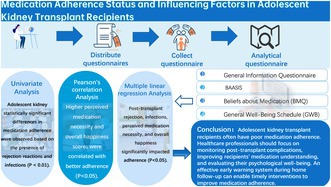

**Abbreviations**
BAASIS: basel assessment of adherence with immunosuppressive medication scale
BMQ: beliefs about medication
GWB: general well‐being schedule

## 1 Introduction

Adolescence, typically defined as the developmental stage from ages 12–18, represents a critical period of physical and mental growth, bridging childhood to adulthood. Adolescent kidney transplant recipients are individuals aged 12 to 18 years who undergo transplantation due to end‐stage kidney disease, with a reported incidence rate of 12.9 per million children [1]. Kidney transplantation is preferred over dialysis, potentially extending life expectancy by 25–30 years post‐operation [2]. Recipients must adhere strictly to lifelong immunosuppressive drug regimens and undergo regular follow‐ups to minimize risks such as acute and chronic rejection, as well as kidney loss. Studies indicate that nonadherence rates among pediatric kidney transplant recipients vary widely, ranging from 30% to 70% [3]. Adolescents, particularly during the critical period of puberty, exhibit a nonadherence rate that is three times higher than that of younger children [4]. A 10% decrease in adherence increases the risk of transplant failure and recipient mortality by 8% [5]. Therefore, early intervention strategies aimed at improving medication adherence among adolescent kidney transplant recipients are crucial for preserving transplant outcomes. Adolescent kidney transplant recipients, navigating a critical period of physical and mental development, often experience complex relationships with parents, feelings of alienation, and challenges in social interactions, potentially leading to anxiety and depression that impact medication self‐management. Various factors influence medication adherence in these adolescents, including cognitive function, social and psychological conditions, family dynamics, and social support [6, 7]. The uniqueness of this study lies in its multidimensional approach. It systematically examined factors influencing medication adherence in adolescent kidney transplant recipients, including general data (such as sociodemographic and disease‐related information), medication beliefs, and overall well‐being, with particular attention to the interaction between physical recovery and psychological experiences. Additionally, we have specifically focused on the transitional phase from dependence to independence in adolescents and propose targeted intervention strategies. This multidimensional analytical approach will provide a more comprehensive perspective on understanding medication adherence in this population. This study conducted a recent survey on medication adherence to immunosuppressive drugs among adolescent kidney transplant recipients in China, analyzing factors that impact adherence. The goal is to provide medical professionals with insights to develop effective intervention strategies.

## 2 Methods

### 2.1 Study Design

This study developed a general data questionnaire to capture factors influencing medication adherence following kidney transplantation, including sex, age, educational level, and post‐transplant complications. An immunosuppressive adherence assessment scale was utilized to evaluate medication adherence both pre‐ and post‐intervention, while medication beliefs were assessed to gauge patients' attitudes toward their medications. The overall well‐being scale was used to assess participants' psychological health. Convenience sampling was employed to select research subjects from transplant centers in four tertiary hospitals across Henan, Shanghai, and Guangdong, renowned for their high volume and quality of transplant procedures in China. A multicenter approach was adopted to increase the sample size and subject diversity, enhancing the generalizability of the study findings and reducing bias in the results.

### 2.2 Participants and Setting

Adolescent kidney transplant recipients who underwent regular follow‐up at transplant centers in four tertiary hospitals in Henan, Shanghai, and Guangdong from February 2023 to October 2023 were selected as research subjects via convenience sampling. The inclusion criteria for patients were as follows: (1) were adolescent kidney transplant recipients aged 12–18 years, (2) underwent the first allogeneic kidney transplant from a donor, (3) were clearly conscious, (4) provided informed consent to participate in this study from adolescent kidney transplant recipients or guardians, and (5) had a duration after discharge > 3 months for nonhospitalized adolescent recipients. The exclusion criteria were as follows: (1) the presence of language, hearing, or comprehension impairments; (2) a history of mental illness, which was based on a clear diagnosis of neurological history and mental disorders by qualified professionals, and an inability to cooperate with follow‐up assessment work; and (3) loss to follow‐up during the study period.

### 2.3 Assessments

#### 2.3.1 General Information Questionnaire

The researchers developed a questionnaire that includes nine variables: gender, age, average monthly household income, healthcare payment methods, educational level, schooling status, living arrangements with a primary caregiver, presence of post‐transplantation rejection reactions, and presence of infections.

#### 2.3.2 Basel Assessment of Adherence With Immunosuppressive Medication Scale (BAASIS)

The scale, developed and endorsed by the Leuven Basel Research Group [8], consists of four Likert items and one binary item. It assesses medication adherence among transplant recipients over the previous 4 weeks across four dimensions: adherence to timing, dose, missed doses, and consecutive missed doses. Each item is rated on a 6‐point scale, ranging from “every day” to “never.” The scores for each item are summed to calculate the overall adherence score, which ranges from 4 to 24 points. A score of 4 indicates good adherence, while scores higher than 4 indicate poorer adherence, with higher scores correlating with increased medication nonadherence. The scale demonstrates acceptable internal consistency, with a Cronbach's *α* coefficient of 0.697.

#### 2.3.3 Beliefs About Medication (BMQ)

Lyu et al. [9] translated the Beliefs about Medicines Questionnaire‐Specific (BMQ‐Specific) into Chinese. This questionnaire comprises two dimensions, medication necessity and medication concerns, each containing five items. Responses are rated on a 5‐point Likert scale from “strongly disagree” to “strongly agree,” yielding scores ranging from 5 to 25 for both necessity and concern dimensions. Scores of 15 or higher indicate high levels of necessity or concern, respectively. The total score, ranging from −20 to 20 points, is derived by subtracting the concerns score from the necessity score. A positive score reflects positive medication beliefs, whereas a negative score indicates negative beliefs. Cronbach's α coefficients for the necessity and concern dimensions are 0.743 and 0.860, respectively, indicating acceptable internal consistency. The test–retest reliability was 0.759.

### 2.4 General Well‐Being Schedule (GWB)

The general well‐being (GWB) scale, initially developed by Fazio and later revised by Duan Jianhua [10], includes six dimensions: vitality; concern about health; mood, encompassing depression and pleasure; satisfaction and interest in life; relaxation and tension (anxiety); and control over emotions and behavior, totaling 18 items. Each item's scores are summed to yield a cumulative score, with higher scores indicating greater subjective well‐being. The scale has demonstrated good reliability and validity. Cronbach's α coefficient of the scale is 0.912, indicating high internal consistency. The test–retest reliability was 0.85.

### 2.5 Data Collection

The survey was conducted using questionnaires distributed individually by transplant centers at each hospital through their respective follow‐up platforms. Prior to administering the survey, trained follow‐up personnel thoroughly explained its purpose to adolescent recipients and their parents via telephone and the WeChat platform. After obtaining informed consent, recipients were provided with the questionnaires along with detailed explanations of the items, their meanings, completion procedures, and precautions. Recipients completed the questionnaires independently and submitted them online upon completion. The validity of the questionnaires was assessed by two follow‐up personnel who collaborated to check for omissions, errors, and duplicate entries. The questionnaires were deemed valid if all the items were completed. A total of 121 questionnaires were distributed, and 115 valid questionnaires were collected. Six invalid questionnaires, such as “overly short answers”, “repeated answers” and “submission”, were deleted, and the respondents did not meet the test requirements, for an effective recovery rate of 95.04%.

### 2.6 Statistical Analysis

The statistical analysis utilized SPSS 23.0 software (Manufacturer Information: IBM Corp. Released 2015. IBM SPSS Statistics for Windows, Version 23.0. Armonk, NY: IBM Corp.). Quantitative data with a normal distribution are presented as the means ± standard deviations (±s), whereas categorical data are presented as frequencies and percentages (%). Single‐factor analysis of variance and *t*‐tests were employed to examine medication adherence across different demographic characteristics among adolescent recipients. Pearson correlation analysis was conducted to examine the relationships among medication adherence, the medication belief questionnaire, and the general well‐being questionnaire. Multiple stepwise regression analysis was used to identify the primary factors influencing medication adherence in adolescent kidney transplant recipients, with *p* < 0.05 or *p* < 0.01 considered statistically significant.

## 3 Results

### 3.1 Current Status of Medication Adherence Levels and Related Factors Among Adolescent Kidney Transplant Recipients

The medication adherence score among the 115 adolescent kidney transplant recipients was 4.98 ± 2.32 points, accounting for 60.08% (76 out of 115) of the patients who reported poor adherence (scores > 4) (Table 1).

**TABLE 1:** Medication adherence levels and scores of relevant factors among adolescent kidney transplant recipients (*n* = 115).

	Score
Medication adherence	4.98 ± 2.32
Medication belief	6.00 ± 5.34
Medication necessity	20.30 ± 3.45
Medication concerns	14.30 ± 4.73
Overall well‐being	78.07 ± 17.01

### 3.2 Univariate Analysis of Factors Influencing Medication Adherence Among Adolescent Kidney Transplant Recipients

The results of single‐factor analysis, based on sex, age, average monthly household income, healthcare payment methods, educational level, schooling status, and living arrangement with a primary caregiver, revealed no statistically significant differences in medication adherence scores among adolescent kidney transplant recipients (*p* > 0.05). However, statistically significant differences in medication adherence were observed based on rejection reactions and infections (*p* < 0.01), as detailed in Table 2.

**TABLE 2:** Univariate analysis of factors influencing medication adherence among adolescent kidney transplant recipients (*n* = 115).

Variables	*n* (%)	Score	*t* (or *F*)	*p*
Gender				
Male	68 (59.13)	4.72 ± 1.42	−1.30	0.202
Female	47 (40.86)	5.36 ± 3.19		
Age (years)				
12–14	46 (40.00)	4.44 ± 1.03	2.31	0.104
14–16	40 (34.78)	5.23 ± 2.08		
16–18	29 (25.22)	5.52 ± 2.92		
Average monthly household income (CNY ￥)				
2000–5000	74 (64.35)	4.97 ± 2.33	0.11	0.896
5000–8000	31 (26.95)	5.10 ± 2.64		
> 8000	10 (8.70)	4.70 ± 0.95		
Form of medical expense payment				
Self‐payment	18 (15.65)	5.17 ± 2.28	0.23	0.879
Urban resident basic medical insurance	22 (19.13)	4.68 ± 1.17		
Rural cooperative medical Insurance	70 (60.87)	5.06 ± 2.67		
Other	5 (4.35)	4.60 ± 0.89		
Educational level				
Primary education or blow	24 (20.87)	4.83 ± 2.12	0.65	0.523
Junior secondary education	68 (59.13)	4.87 ± 2.13		
Senior secondary education or above	23 (20.00)	5.48 ± 3.03		
Schooling situation				
Boarding	9 (7.83)	5.44 ± 3.00	0.58	0.559
Day student	92 (80.00)	5.02 ± 2.42		
Other	14 (12.17)	4.43 ± 0.65		
Living with a primary caregiver				
Yes	114 (99.13)	4.99 ± 2.34	0.00	0.994
No	1 (0.87)	5.00		
Acute rejection				
Yes	10 (8.70)	6.80 ± 4.49	−2.66	0.009
No	105 (91.30)	4.81 ± 1.96		
Infection				
Yes	5 (4.35)	7.20 ± 5.01	−3.29	0.001
No	110 (95.65)	4.77 ± 1.80		
Post‐transplant clinical course				
< 12 months	15 (13.04)	4.33 ± 0.72		
12–36 months	44 (38.26)	5.36 ± 3.19	0.529	0.851
> 36 months	56 (48.70)	4.86 ± 1.69		

### 3.3 Correlation Analysis of Medication Adherence, Medication Necessity, Medication Concerns, and Overall Well‐Being Among Adolescent Kidney Transplant Recipients

Pearson's correlation analysis results indicated that higher scores for medication necessity and overall well‐being were associated with lower medication adherence scores among adolescent recipients, suggesting improved adherence (all *p* < 0.05), as shown in Table 3.

**TABLE 3:** Pearson correlation analysis of medication adherence with medication belief and overall well‐being.

	Medication necessity	Medication concerns	Overall well‐being	Medication adherence
Medication necessity	1			
Medication concerns	0.177	1		
Overall well‐being	0.074	−0.166	1	
Medication adherence	−0.230*	−0.055	−0.267**	1

*Note:* Significance codes: *P < 0.05; **P < 0.01.

### 3.4 Multifactor Analysis of Factors Influencing Medication Adherence Among Adolescent Kidney Transplant Recipients

Using the stepwise method, multiple linear regression analysis was conducted to explore the factors influencing medication adherence among adolescent kidney transplant recipients. Medication adherence was considered the dependent variable, while independent variables included factors with statistical significance (*p* < 0.05) from the single‐factor analysis, along with the significant variables of medication necessity and overall well‐being identified in the correlation analysis. The results indicated that post‐transplant rejection reactions, infections, medication necessity, and overall well‐being were significant predictors in the regression equation, as detailed in Tables 4 and 5.

**TABLE 4:** Independent variable assignment.

Independent variables	Scoring method
Rejection	0 = Not Present, 1 = Present
Rejection	0 = Not Present, 1 = Present
Medication necessity	Original Value Entry
Overall well‐being	Original Value Entry

**TABLE 5:** Regression analysis results of factors influencing medication adherence among adolescent kidney transplant recipients.

Variable	Coefficients	Standard error	Standardized coefficients	*t*	*p*
Constant	7.461	2.006	—	3.718	< 0.001
Rejection	1.837	0.633	0.224	2.900	0.005
Infection	4.534	0.893	0.400	5.080	< 0.001
Medication necessity	−0.114	0.051	−0.170	−2.248	0.027
Overall well‐being	−0.030	0.014	−0.168	−2.180	0.031

## 4 Discussion

The findings of this study revealed that the medication adherence score among adolescent kidney transplant recipients was 4.98 ± 2.32 points, with a considerable proportion of patients demonstrating poor adherence (60.08%, 76 out of 115). Among the 60.08% of adolescent kidney transplant recipients with poor medication adherence, 5 children experienced graft loss, with a 3‐year survival rate of 93.42% (71/76). In contrast, none of the 39.92% of the recipients with good adherence experienced graft loss. The incidence of rejection in recipients with good medication adherence was 28.21% (11/39), whereas in those with poor medication adherence, it was 32.89% (25/76). These findings indicate that children with poor medication adherence have a greater incidence of rejection. Adolescent recipients display varying degrees of medication nonadherence, which may correlate with feelings of social isolation and differences, potentially contributing to an elevated rate of treatment nonadherence [11]. However, medication adherence among patients did not significantly differ concerning sex, age, educational level, or average monthly household income (all *p* > 0.05). A study on medication adherence among adolescents with depression [12] also revealed no significant differences in medication adherence among patients of different ages, sexes, and places of residence, which further supports the notion that sex and age may not be key factors affecting adherence. The study by Boucquemont et al. [6], which conducted an in‐depth analysis of medication adherence among adolescent and young adult kidney transplant recipients, concluded that there was no significant difference between gender and medication adherence. This finding further supports our study's conclusion that no significant difference exists between gender and medication adherence among adolescents. This finding contrasts with the findings reported by Yang et al. [13], who studied the impact of different factors on medication adherence among adult kidney transplant recipients. The divergence may arise from the specific focus of the current study on adolescents aged 12–18 years, a group that continues to rely on caregivers to manage their health. During this developmental stage, factors such as educational level, schooling status, and living arrangements with caregivers remain relatively stable, potentially exerting a lesser influence on medication adherence.

Single‐factor analysis revealed that rejections and infections significantly impact medication adherence among adolescent kidney transplant recipients, which aligns with the findings reported by Wang et al. [14] In adult follow‐up kidney transplant recipients, this may be attributed to the increase in complications, increasing the risk of disease exacerbation and resulting in physical discomfort. Furthermore, adolescents experience a sensitive period during which illness recurrence often triggers varying levels of anxiety and depression, and complications can stress the mental health of adolescents, especially given the complexity of medication regimens and frequent adjustments, affecting their attitudes and beliefs toward medication [15], potentially leading to medication nonadherence. It can be seen that there is a complex bidirectional relationship between medication adherence and medical complications, which deserves the close attention of medical staff.

Horner et al. [16] contend that an individual's medication beliefs shape his or her medication behavior. Research indicates that adolescent recipients with higher medication scores exhibit better medication adherence. Adolescents perceive a greater need for medication and harbor relatively fewer concerns, suggesting that the majority of adolescent recipients recognize the significance of lifelong adherence to immunosuppressive drugs and maintain a positive attitude toward medication. This perception of medication importance and necessity is crucial, as adherence to immunosuppressive therapy can prevent rejection and infection following transplantation, thereby ensuring the long‐term success of the graft and the overall health of the recipient. Studies have indicated [17] that cognitive development levels and behavioral habits also affect children's medication adherence. Younger children may require more guidance and support to take medication correctly. Scholars have developed structured transition programs that cover four areas: awareness, insight, self‐reliance, and the establishment of healthy habits, which can help children transition smoothly into adulthood [18].

The study findings demonstrate a positive correlation between medication adherence and overall well‐being in adolescent kidney transplant recipients, indicating that individuals with higher levels of positive emotions are more likely to adhere to their medication regimen. Adolescents undergoing kidney transplantation experience a critical phase of physical and mental development, confronting challenges typical of adulthood, such as transitioning from school to work, forming romantic relationships, and gaining independence from their parents. Moreover, they are transitioning from pediatric to adult healthcare systems, experiencing complex dynamics with their parents, and encountering social integration difficulties with peers, all of which can affect their ability to manage medications [19]. As a result, follow‐up nurses should assess their psychological well‐being and consider how caregivers' post‐traumatic growth influences their physical and mental recovery [20]. Tailored and multimodal psychological interventions should be implemented, incorporating targeted positive psychological strategies such as gratitude journals, building supportive relationships, and mindfulness meditation [21]. These interventions aim to promote positive emotional development, enhance overall well‐being, and mitigate the impact of negative emotions on medication adherence.

Adolescence represents a critical transition period from childhood to adulthood, demanding that adolescents actively learn and master self‐management skills instead of relying solely on caregivers. Gilleland et al. [22] proposed that “transition” encompasses two levels: process and event. The process refers to the gradual shift of healthcare responsibilities from parents to adolescents and young adults (AYAs). In contrast, the event refers to the specific actions involved in transferring from pediatric to adult healthcare teams. While family support significantly influences adolescents' preparation and quality of life during this transitional phase [23], it can also potentially reduce their motivation for active self‐management, leading to neglect and dependency. Therefore, healthcare professionals should consistently stress the importance of medication adherence throughout the follow‐up process, foster positive attitudes and beliefs about medications, and actively provide family health education geared toward transitioning to adult care models. This education should help caregivers balance disease management by promoting independence, empowering adolescents to acquire self‐care skills, take charge of their health, and smoothly transition to adult self‐care practices.

Additionally, most postoperative follow‐up visits for adolescent recipients follow adult models that lack specificity. It can be inferred that establishing dedicated follow‐up processes or gradually introducing adolescent transition clinics would ensure continuous diagnosis and treatment. Introducing personalized and targeted guidance is expected to improve medication adherence behaviors.

## 5 Conclusion

Poor medication adherence is prevalent among adolescent kidney transplant recipients and is influenced primarily by factors such as medication necessity and overall well‐being. Healthcare professionals should prioritize assessing major postoperative complications, understanding the importance of medications, and evaluating the positive psychological status of these recipients during follow‐up. Developing a tailored follow‐up approach for adolescents and implementing specific positive intervention strategies are crucial to improving medication adherence and enhancing long‐term prognostic outcomes. This study has several limitations. First, it is important to acknowledge that relying on self‐reports may introduce biases stemming from the participants' memory and emotional filters. Furthermore, there is a lack of objective clinical data available for analysis, and recall bias may impact the accuracy and reliability of the data. However, self‐reports from patients directly reflect their awareness of their own illness, which allows for an assessment of the current state of medication adherence. Second, these results were obtained from four single centers, so the number of adolescent kidney transplant recipients was small. Given the overall small number of children who underwent kidney transplantation nationwide (about 2000 patients), the sample size was not large, so bias was inevitable. In future studies, we can expand multicenter and multiregional longitudinal surveys and conduct qualitative research to explore the actual experience of these studies further and provide a theoretical basis for the construction of corresponding interventions.

## Author Contributions

**Wenyu Zhao** and **Xiaoying Lu:** conceptualization, visualization, and writing – review and editing; **Yanhua Li** and **Yutong Chen:** conceptualization, formal analysis, and writing – original draft; **Shijun Pu** and **Suxia Yang:** data curation and formal analysis; **Yazhe Duan:** investigation and methodology; **Li Zeng:** writing – review and editing.

## Ethics Statement

All procedures carried out in this study were in accordance with the ethical standards of the institutional and national responsible committee on human experimentation and the Helsinki Declaration of 1964 and its later amendments or equivalents. This study was approved by the Ethics Committee of the First Affiliated Hospital of Naval Medical University.

## Consent

Consent was obtained from all individual patients included in the study.

## Conflicts of Interest

The authors declare no conflicts of interest.

## Data Availability

The data are available upon reasonable request.
